# Absence of Detectable XMRV and Other MLV-Related Viruses in Healthy Blood Donors in the United States

**DOI:** 10.1371/journal.pone.0027391

**Published:** 2011-11-14

**Authors:** Shixing Tang, Jiangqin Zhao, Mohan Kumar Haleyur Giri Setty, Krishnakumar Devadas, Durga Gaddam, Ragupathy Viswanath, Owen Wood, Panhe Zhang, Indira K. Hewlett

**Affiliations:** Lab of Molecular Virology, Center for Biologics Evaluation and Research, Food and Drug Administration, Bethesda, Maryland, United States of America; University of Florida, United States of America

## Abstract

**Background:**

Preliminary studies in chronic fatigue syndrome (CFS) patients and XMRV infected animals demonstrated plasma viremia and infection of blood cells with XMRV, indicating the potential risk for transfusion transmission. XMRV and MLV-related virus gene sequences have also been detected in 4–6% of healthy individuals including blood donors in the U.S. These results imply that millions of persons in the U.S. may be carrying the nucleic acid sequences of XMRV and/or MLV-related viruses, which is a serious public health and blood safety concern.

**Methodology/Principal Findings:**

To gain evidence of XMRV or MLV-related virus infection in the U.S. blood donors, 110 plasma samples and 71 PBMC samples from blood donors at the NIH blood bank were screened for XMRV and MLV-related virus infection. We employed highly sensitive assays, including nested PCR and real-time PCR, as well as co-culture of plasma with highly sensitive indicator DERSE cells. Using these assays, none of the samples were positive for XMRV or MLV-related virus.

**Conclusions/Significance:**

Our results are consistent with those from several other studies, and demonstrate the absence of XMRV or MLV-related viruses in the U.S. blood donors that we studied.

## Introduction

Xenotropic murine leukemia virus-related virus (XMRV) was originally identified in prostate cancer tissues in 2006 [1], and proposed to be associated with PC [1], [2], [3], [4], [5] and chronic fatigue syndrome (CFS) [6], [7]. However, a causal relationship has not been validated and several controversial findings have been reported [8], [9], [10], [11], [12]. Furthermore, XMRV as a human pathogen has been questioned since mouse DNA contamination has been found in human samples tested [13], [14], [15], [16], and XMRV may be the result of a recombination of two MLV ancestors [17]. As a newly identified retrovirus, XMRV can infect human tissues and cells including lymphoid organs [18] and peripheral blood mononuclear cells (PBMCs) [6], indicating potential transfusion transmission of XMRV. XMRV has also been detected in 3.7% of healthy individuals [6] and 5.9% of non-prostate cancer patients [2] in the U.S.. In addition, Lo et al reported that 6.8% of U.S. healthy blood donors carried MLV-related sequences, which are molecularly different from but very similar to XMRV [19]. These results, if confirmed, imply that millions of persons in the U.S. may harbor XMRV and/or MLV-related viruses and thus pose a serious threat to public health, including blood safety and organ transplantation. To ensure blood safety, suggestions and preventive measures have been proposed, such as developing screening tools and deferring CFS patients for blood donation [20]. However, these recommendations and measures have been questioned in the absence of the conclusive consensus of the prevalence of XMRV infection in blood donors and causality for human diseases. In order to address blood safety concerns, the Blood XMRV Scientific Research Working Group (SRWG) composed of members from academia, government and blood organizations was formed by the National Heart, Lung, and Blood Institute (NHLBI) [21]. The major goals of this group were to 1) validate the testing methods for XMRV since one of the possible reasons for the conflicting findings was attributed to differences in testing methods, and 2) to investigate possible infection of blood donors with XMRV or MLV-related viruses.

During the past two years, our laboratory actively participated in assay validation and assessment of the threats posed by XMRV on blood safety. We previously reported that our RT-PCR assay could detect 10 copies and 1 copy of plasmid DNA in the 1^st^ and 2^nd^ round PCR, respectively [22] by using primers described by Silverman et al [1] and Mikovits et al [6]. Our quantitative PCR assay could detect 1–10 copies of XMRV plasmid DNA, which is comparable to the results reported by Schlaberg et al [2]. Our PCR assays were able to achieve similar levels of sensitivity and specificity based on the spiked XMRV panels created by the Blood XMRV SRWG [21]. For virus culture, we set up an infectivity assay using the Detectors of Exogenous Retroviral Sequence Elements (DERSE) indicator cells where plasma samples are co-cultured with modified LNCaP cells which are susceptible to XMRV infection and virus replication monitored using a fluorescence signal [23]. Mikovits et al who reported the association of XMRV with CFS claimed that culture of virus from plasma was the most sensitive blood-based assay for detection of XMRV [7]. By using these highly sensitive assays, we screened U.S. blood donors for XMRV or MLV-related viruses in order to provide further evidence of the status of these possible new viruses in the blood donors from the NIH Blood Bank, the same blood bank from which donors had previously reported to harbor polytropic MLV-related virus sequences in 6.8% of the individuals tested [19].

## Materials and Methods

### Ethics Statement

The Food and Drug Administration Research Ethics Committee has waived the need for consent due to the fact the blood donor material used was fully anonymised.

### Collection and PCR testing

A total of 71 PMBC samples and 110 plasma samples from blood donors were enrolled in our study. Both plasma and PBMCs were recovered from the entire buffy coat that was received from the NIH Blood Bank. Briefly, the entire buffy coat was centrifuged at 1500 rpm for 15 minutes and plasma was carefully removed. Cells were resuspended in 15 ml of Ficoll solution and centrifuged for 30 minutes at 400g. The PBMCs, seen as a ring or band at the top of the Ficoll solution, were removed, placed in a fresh 50 ml tube and filled with PBS saline for further use.

Viral RNA was extracted from 140 µl of plasma using QIAamp MiniElute Virus Spin kit (Qiagen, Valencia, CA), and genomic DNA of 1×10^6^ PBMCs was extracted using the QIAamp DNA Blood mini kit. Reverse transcription was performed with SuperScript III for First-strand Synthesis System (Invitrogen) using 8 ul of viral RNA or total nucleic acid from PBMC and XMRV gag reverse primer 1154R [6]. For amplification of XMRV gag gene, first-round PCR was performed in a 20 ul volume containing 5 ul of cDNA or 200∼500 ng of genomic DNA, 10 ul of 2xPCR buffer (Extensor Hi-Fidelity ReddyMix PCR Master Mix, ABgen House, Surrey, UK) and 2.5 pmol each primer (GAG-O-F and GAG-O-R) [1]. Reaction conditions were one cycle at 94°C, 5′, 45 cycles at 94°C, 1′, 58°C , 1′, 72°C, 1′ and one cycle at 72°C, 7′. Two microliters of 1^st^ round PCR products were added to 2^nd^ round PCR with the same reaction conditions as those in the 1^st^ PCR except that the different primers (GAG-I-F and GAG-I-R) and the annealing temperature of 60°C were used [1]. Each PCR run included both XMRV positive control (a full-length XMRV plasmid DNA, isolate VP62, gifted by Dr R. Silverman) and negative control (water). PCR amplification products were visualized on a 2% agarose gel stained with ethidium bromide. Each sample was tested in triplicate, the band equivalent to the correct size of positive control was excised from 2% agarose gel using the QIAquick gel extraction kit (Qiagen Inc., Valencia, CA) for sequence analysis. Alternatively, a specific PCR product was purified using ExoSAP-IT reagent (usb, Santa Clara, CA). Purified PCR products were sequenced directly using the ABI Prism BigDye Terminator Cycle Sequencing kit in the ABI PRISM 310 Genetic Analyzer (Applied Biosystems, Foster City, CA). Sequence and phylogenetic analyses were performed using the MEGA5 software package and the Invitrogen Vector NTI software, version 11.3.0 (Invitrogen, Carlsbad, CA). A positive test result was defined as one where at least one band of the correct size was detected in triplicate PCR reactions, and confirmed by sequencing as XMRV. A negative result was defined as one where no bands of the correct size were detected in triplicate PCR reactions or at least one band of correct size was observed but the sequence analysis did not confirm as XMRV. To ensure integrity of extracted DNAs, human GAPDH gene was amplified with the same PCR primers (hGAPDH-66F and hGAPDH-291R) and conditions published previously [1]. To avoid possible mouse DNA contamination, PCR assays for amplifying mouse intracisternal A particle (IAP), mouse mitochondrial DNA were performed as previously described [13], [15], [19]. The experiments were performed by two laboratory personnel to ensure that results were scored based on reproducibility of data obtained by two independent operators.

### Cell culture assay for detection of infectious virus

A co-culture assay was adopted to monitor XMRV infection by using Detectors of Exogenous Retroviral Sequence Elements (DERSE) cells that are LNCaP-iGFP cell clones displaying sensitivity to XMRV infection that leads to expression of a GFP reporter [23]. In this assay, a derivative of LNCaP cells termed DERSE.LiGP cells (a gift from Dr Vineet KewalRamani, NCI) were used. DERSE cells were selected to express pBabe.iGFP-puro, a MLV proviral vector encoding an intron-interrupted GFP reporter gene. In this indicator cell line, GFP is only expressed after mobilization by an infecting gammaretrovirus during a second round of infection. Briefly, 0.4×10^5^ DERSE cells/well were added in 24-well plate. After 24–48 hours, the cells were mixed with 200 ul of plasma samples or normal plasma spiked with XMRV. The plate was centrifuged at 1500 rpm (Eppendorf Centrifuge # 5810 R) for 5 minutes, and then incubated at 37°C overnight. Plasma was very carefully replaced with fresh RPMI complete media, and transferred to a 6-well plate to expand as required (usually after 4–5 days post infection). When cells became confluent, they were transferred to a T-25 flask and maintained for 21 days post infection. GFP expression in cells at different days post-infection was determined using fluorescence microscopy.

## Results

**Figure 1:**
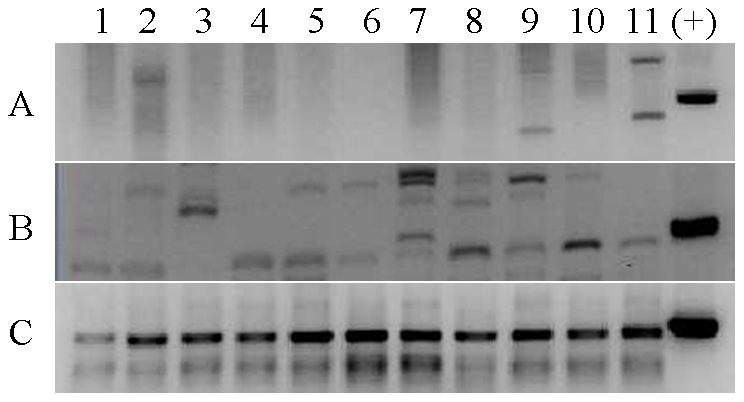
PCR screening for XMRV or MLV-related virus. (A) PCR products of 11 plasma samples (lane 1–11) collected in NIH Blood Bank with XMRV gag gene primer pair. Lane 12 was positive control of XMRV. (B) PCR products of 11 PBMC samples (lane 1–11) collected in the NIH Blood Bank with XMRV gag gene primer pair. Lane 12 was positive control of XMRV. (C) hGAPDH gene. Lane 1–11 was results for 11 PBMC samples while lane 12 was positive control for hGAPDH.

By using serial 1∶10 dilutions of XMRV plasmid DNA with known copy numbers based on absorbance A260 of the purified plasmid VP62, 10 copies and one copy of plasmid DNA were detected in the first- and second-round PCR, a lower detection limit of one copy of proviral DNA using our current nested PCR conditions was achieved. The sensitivity of the PCR assays was also evaluated using XMRV DNA extracted from a series of 1∶10 dilutions of 22Rv1 cells (CRL-2505, ATCC, Gaithersburg, MD) that harbor multiple copies of integrated XMRV provirus and constitutively produce infectious virus [24]. The current nested PCR assay could detect XMRV DNA from single 22Rv1 cells (data not shown). Using this assay, none of the 110 plasma samples were positive for XMRV or MLV-related virus with either XMRV gag primer sets although the positive control was successfully amplified in each PCR run (Fig. 1A). Total nucleic acid from 71 PBMC samples was also tested but found to be negative for XMRV or MLV-related virus using both nested DNA PCR and RT-PCR assays (Fig. 1B, Table 1). Both assays were used since it was reported that RT-PCR could be more sensitive than DNA PCR for detection of XMRV in activated PBMCs [7]. Any bands with similar size of XMRV positive control were excised from the gel, purified and sequenced. No XMRV sequences were found on sequence analysis. A specific hGAPDH gene was amplified from all 71 PBMC samples (Fig. 1C), indicating the integrity of the extracted DNA.

**Table 1:** Detection of XMRV in the plasma and PBMC samples from the NIH Blood Bank^1^.

Sample	PCR results		DERSE results	
	No. tested	No. positive	No. tested	No. positive
Plasma	110	0	33	0
PBMCs	71	0	0	0

1 Viral RNA isolated from plasma was analyzed for XMRV and HIV-1 using RT-nested PCR while genomic DNA extracted from PBMCs was analyzed for XMRV and HIV-1 using nested PCR and (q)PCR.

GAPDH was amplified in parallel as an internal control.

**Figure 2:**
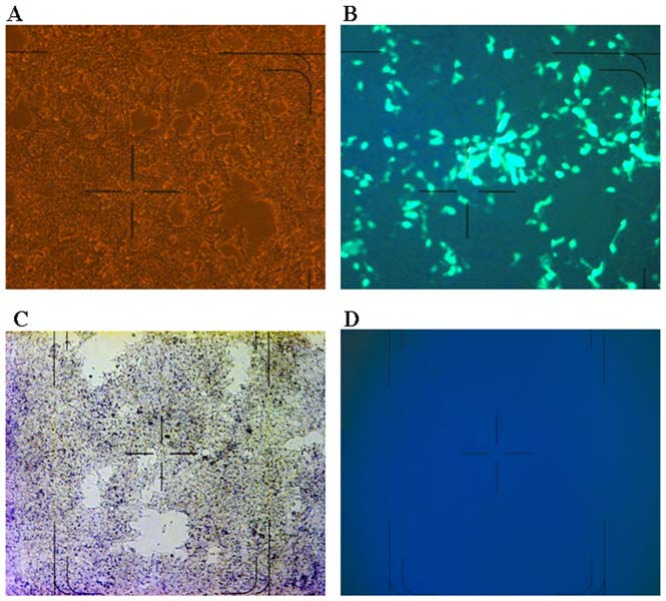
GFP signal detection in DERSE cell culture. (A) Light microscopy image for positive control. DERSE cells were infected with culture supernatant from 22Rv1 cell. (B) Fluorescence microscopy image for XMRV positive control. Panel C (light microscopy image) and D (fluorescence microscopy image) for blood donor plasma in which no XMRV was detected.

Using DERSE cells, the GFP signal could be detected within three days of XMRV infection, with the number of GFP-positive cells increasing over subsequent days. The DERSE GFP culture method is highly sensitive as it can detect around 2000 copies of XMRV. In our study, DERSE cells could be successfully infected by culture supernatant of the 22Rv1 cell line which carries XMRV [24] (Fig. 2A) and displayed fluorescence 4 days after infection (Fig. 2B). GFP expression was observed 18 days post infection in cells that were infected with 2000 copies of XMRV. However, none of the 33 plasma samples tested displayed visible fluorescence signal even after 21 days post infection (Fig. 2D). The culture supernatants were also negative for XMRV using both quantitative PCR and RT-PCR (data not shown).

## Discussion

The above results strongly support the conclusion that XMRV and other MLV-related viruses are absent in healthy blood donors in the population we studied. The rigorous testing employed and use of highly sensitive PCR and cell culture methods to evaluate the presence of both nucleic acid and infectious virus provide strong evidence to support this conclusion. The failure to detect XMRV in U.S. blood donor samples is unlikely due to the sensitivity of PCR assays because they have been shown to be at least as sensitive as those previously reported [22], and comparable to those used by other labs enrolled in the assay evaluation study sponsored by the Blood XMRV SRWG [21]. XMRV positive and negative controls were correctly identified in both PCR and co-culture experiments in our study indicating the accuracy of test performance and validity of assay runs. In addition, the sample size we tested was sufficiently large enough to potentially identify at least 3–4 XMRV or MLV-related virus positive samples since between 4–6% of healthy controls including blood donors were reported to be positive for XMRV or MLV-like viruses in previous studies conducted in the U.S. [2], [6], [19]. Therefore, based on testing using highly sensitive detection assays we did not find evidence of XMRV or MLV-related virus infection in the U.S. blood donor samples we tested.

Our results are consistent with other recent findings that have been reported in the U.S. Gao et al tested 425 plasma samples from U.S. blood donors using a transcription mediated amplification (TMA) assay and did not detect XMRV in these samples [25]. Their assay was reported to be one of the most sensitive assays in the assay evaluation study sponsored by the Blood XMRV SRWG [21]. Qiu et al reported that only 0.1% of the U.S. blood donors were positive for anti-XMRV antibodies by using their prototype direct chemiluminescent immunoassays (CMIAs) on the automated ARCHITECT® instrument for detecting anti-XMRV assay, which is the first immunoassay that has been evaluated by the well characterized XMRV infected animal bleeds [26]. Switzer et al were unable to detect XMRV infection in 51 healthy controls and 43 U.S. blood donors using PCR and serology assays [27]. Kunstman et al tested 996 samples from the Chicago Multicenter AIDS Cohort Study (562 HIV-1 positive and 434 at high risk for HIV-1 infection, but HIV-1 negative individuals), none of them were XMRV positive [28]. Henrich et al were unable to detect XMRV infection in PBMC samples from 43 HIV positive individuals, 97 rheumatoid arthritis patients, 26 transplant recipients and 95 general patients [29].

XMRV was also not or rarely detected in general populations worldwide. Only about 1% of control groups were found to be positive for XMRV in Germany [10], the U.K [12] and Japan [30], but no XMRV was detected in Chinese blood donors [31]. Negative results were reported for XMRV testing of blood donors or individuals infected HIV-1 in Africa [22]. These results indicate that XMRV or other MLV-like viruses may be very rare, or absent in the general population overall. In contrast, our results support the recent findings that the current positive detection of XMRV or MLV-related virus in human samples may be due to mouse DNA contamination rather than a true human infection. Robinson et al reported that XMRV positive prostate cancer tissues and 21.5% of XMRV negative cases were positive for mouse IAP sequence [15]. Oakes et al found that by using a less specific PCR assay, both XMRV and/or MLV were detected in CFS patients. However, all positive samples were also positive for mouse IAP while no contamination was observed in any of the negative control samples [13]. Sato et al reported that endogenous MLV was amplified in a commercial RT-PCR kit using standard primers for XMRV [16]. The contamination originated from the hybridoma cell line from which the monoclonal antibody used in the polymerase reaction mixture to facilitate hot-start PCR was prepared. Hue et al also demonstrated that XMRV specific primers can amplify murine endogenous viral sequences [14]. These results indicate that mouse DNA contamination is widespread and can confound XMRV detection in human samples.

Furthermore, Hue et al compared the published XMRV sequences with those from 22Rv1 cell, which is infected with XMRV and found that the genetic distance among 22Rv1-derived sequences exceeds that of patient-associated sequences, indicating that patient-associated XMRV sequences are consistent with laboratory contamination rather than a true human infection [14]. The 22Rv1 cell line was derived from a human prostate cancer xenograft (CWR22) that was serially passaged in nude mice in 1990s. Interestingly, it was recently shown by Paprotka et al that XMRV resulted from recombination between two endogenous MLVs during passage of the CWR22 PC xenograft [17], suggesting that the laboratory-derived virus may have contaminated samples for more than a decade and thereby contributed to the inconsistent positive detection reported by various laboratories that had used them for these studies and over extended periods of time. The relevant published studies on XMRV and MLRV findings in CFS, PCA and blood donors are listed in the Table S1.

In summary, we screened 110 plasma samples and 71 PBMC samples collected from U.S. blood donors using well characterized and highly sensitive PCR and culture assays. The testing employed independent test operators and rigorous testing conditions aimed at avoiding contamination. Under these conditions, none of the samples were found to be positive for XMRV or MLV-related virus sequences or infectious virus. Our results failed to demonstrate the presence of XMRV or MLV-related viruses in the samples we tested, and provide strong evidence for the absence of XMRV or MLV-related virus in the U.S. blood donor population we studied.

## Supporting Information

Table S1 **Relevant Published Studies on XMRV and MLRV Findings in CFS, PCA and Blood Donors.**
(DOC)
