# Supplementary material for: Absence of Detectable XMRV and Other MLV-Related Viruses in Healthy Blood Donors in the United States
Source: PLoS One. 2011 Nov 14;6(11):e27391. doi: 10.1371/journal.pone.0027391 (PMC3215715; doi:10.1371/journal.pone.0027391)
Supplement: Table S1 — Relevant Published Studies on XMRV and MLRV Findings in CFS, PCA and Blood Donors. (DOC) [file pone.0027391.s001.doc]

**Table 1. Relevant Published Studies on XMRV and MLRV Findings in CFS, PCA and Blood Donors**

| **Study** | **Samples** | **Collected**  **Country** | **Described Assays**  **Methods** | **Results** | |
| --- | --- | --- | --- | --- | --- |
|  |  |  |  | **Sample detection (%)** | **XMRV test** |
| **Prostate Cancer Studies** | | | | | |
| Urisman A, Molinaro RJ, Fischer N, Plummer SJ, Casey G, Klein EA, et al.. PLOS. 2006; 2(3): 211-225. | 86 PCA tissue samples | United States | TRIZOL reagent, PCR, TAQMAN genotyping assay, microarray screening, FISH, cloning, sequencing, | 8/20 (40%) RNASEL R462Q- homozygous cases  0/14 (0.0%) heterozygous  1/52 (1.9%) homozygous wild-type (familial PCA) | positive |
| Fischer N, Hellwinkel O, Schulz C, Chun FKH, Huland H, Aepfelbacher M, et al. J Clin Virol. 2008; 43:277-283. | 105 PCA tissue samples from 87 patients  70 tissue controls | Germany | RNA  Nested RT-PCR | 1/105 (0.95%) positive  (non-familial PCA)  1/70 (1.42%) positive  (healthy prostate tissue) | positive |
| Hohn O, Krause H, Barbarotto P, Niederstadt L, Beimforde N, Denner J, et al. Retrovirology. 2009; 6(1): 92. | 589 PCA tissue samples  146 PCA serum samples | Germany | DNA/RNA gag  Nested RT-PCR  ELISA | 0/589 (0.0%) DNA positive  0/146 (0.0%) Ab positive | negative |
| Schlaberg R, Choe DJ, Brown KR, Thaker HM,Singh IR. PNAS. 2009; 106 (38): 16351-16356. | 233 PCA tissue samples  101 tissue controls | United States | Tissue, DNA  Quantitative PCR  XMRV-specific stain IHC | 14/233 (6.2%) PCA PCR  54/233 (23.0%) IHC stain  2/101 (2.0%) Controls PCR  4/101 (4.0%) IHC stain | positive |
| Arnold RS, Makarova NV, Osunkoya AO, Suppiah S, Scott TA, Johnson NA, et al. Urology. 2010; 75 (4): 755-761. | 40 PCA plasma samples | United States | Serological assay (Neutralizing antibodies). IHC, FISH, nested PCR | 11/40 (27.5%) sera positive for neutralizing anti-XMRV antibodies.  8/20 (40%) RNASEL QQ  3/20 (15%) RNASEL RQ or RR  Consistent with PCR and FISH results | positive |
| **Chronic Fatigue Syndrome Studies** | | | | | |
| Lombardi VC, Ruscetti FW, Das Gupta J, Pfost MA, Hagen KS, Peterson DL, et al. Science. 2009:326; 585-589. | 101 CFS samples  218 controls | United States | PBMCs, DNA  Gag Nested PCR  WB  WPI Lab | 68/101 (67.0%) CFS positive  8/218 (3.7%) controls positive  OR = 54.1(95%CI: 23.8 -122) | positive |
| Erlwein O, Kaye S, McClure MO, Weber J, Wills G, Collier D, et al. PLoS ONE. Jan 2010; 5(1); e8519. | 186 CFS samples | United Kingdom | DNA for XMRV and MLV. Nested PCR. Assay controls used. | 0/186 (0.0%) CFS | negative |
| Groom HC, Boucherit VC, Makinson K, Randal E, Bapista S, Hagan S, et al. Retrovirology. 2010; 7(10). | 170 CFS samples  395 controls | United Kingdom | PBMCs. XMRV DNA. Real time PCR (gag and env)  RT-PCR  Viral neutralization assay for XMRV and MLV. | 0/170 (0.0%) CFS PCR  1/28 (3.6%) CFS serum showed XMRV neutralizing activity.  0/395 (0.0%) Controls PCR  25/395 (6.3%) positive for neutralizing assay.  Strong cross reactive with VSV. | positive |
| Van Kuppeveld FJM, de Jong AS, Lanke KH, Verhaegh GW, Melchers WJG, Swanink CMA, et al. BMJ. 2010; 340: c1018. | 32 CFS samples.  43 controls  A matched case-control study | Netherlands | PBMCs. DNA.  Nested PCR of gag gene.  Real time PCR of integrase gene | 0/32 (0.0%) CFS  0/43 (0.0%) controls | negative |
| Lo SC, Pripuzova N, Li B, Komaroff AL, Hung GC, Wang R, et al. PNAS. 2010. 107(36): 15874-9. | 37 CFS PBMC samples  44 blood donor controls | United States | Gag Nested PCR  MLV  RT-PCR | 32/37 (86.5%) PBMC CFS  3/44 (6.8%) PBMC  controls | positive |
| **Other Diseases/Populations** | | | | | |
| Henrich TJ, Li JZ, Felsenstein D, Kotton CN, Plenge RM, Pereyra F, et al. J Infect Dis. 2010: 202; 1478-1481. | 32 CFS PBMCs  43 HIV positive cryopreserved PBMCs  97 RA cryopreserved PBMCs  26 transplant recipients cryopreserved PBMCs  95 general patients cryopreserved PBMCs | United States | PBMC DNA PCR (Qiagen) | 0/32 (0.0%) CFS  0/43 (0.0%) HIV  0/97 (0.0%) RA  0/26 (0.0%) transplant recip.  0/95 (0.0%) controls | negative |
| Kunstman KJ, Bhattacharya T, Flaherty J, Phair JP, Wolinsky SM. AIDS. 2010; 24(11): 1784-1785. | 996 men from the Chicago Multicenter AIDS Cohort Study  562 HIV+ ; 434 at risk, HIV- | United States | qPCR for *gag* sequences | 0/562 (0.0%)  0/434 (0.0%) | negative |
| Tang S, Zhao J, Viswanath VR, Nyambi PN,Redd AD, Dastyar A, Spacek LA, et al. Transfusion. 2010;51(3):463-468 | 199 plasma HIV pos  19 PBMNC HIV pos  50 culture supernatants from PBMNCs | Cameroon  Uganda | RT PCR DNA on plasma  Real time PCR on PBMCs  qPCR | 0/199 (0.0%) HIV plasma  0/19 (0.0%) HIV PBMNC  0/50 (0.0%) culture supernatants | negative |
| **Blood Donor Studies** | | | | | |
| Japanese Study; Furuta et al, Cold Spring Harbor symposium, 2009 | blood donors | Japan | Serum, Western blot | 1.0 – 3.0% Ab pos in blood donors |  |
| Gao et el. (Gen-Probe and ARC) 2010 International Conference on Emerging Infectious Diseases; July 13, 2010, Atlanta GA | 1435 plasma US blood donors  44 HIV-1 positive samples | United States | TMA; DNA and RNA;  research assay TIGRIS system | 0/1435 (0.0%) blood donors  0/44 (0.0%) HIV-1 positives | negative |
| Hong P, Li J, Li Y. et al.. Virology Journal. 2010; 7:224. doi:10.1186/1743-422X-7-224. | 65 CFS patients  85 blood donors  (65 healthy; 20 with HBV, HCV, HIV and/or HTLV) | China | RT PCR  PBMCs and plasma | 0/65 (0.0%) CFS  0/65 (0.0%) healthy blood donors  The other 20? | negative |
| Switzer W, Jia H, Hohn O, Zheng H, Tang S, Shankar A, Bannert N, et al., Retrovirology. 2010;7:57. | 51 CFS cases  53 healthy controls  41 U.S. blood donors | United States | Nested PCR, WB, ELISA, IFA  Tested in three labs | 0/51 (0.0%) CFS  0/53 (0.0%) controls  0/41 (0.0%) blood donors | negative |

**Abbreviations:** **CFS** – chronic fatigue syndrome; **COPD** – chronic obstructive pulmonary disease; **CSF** – cerebral spinal fluid; **FISH** – fluorescence *in situ* hybridization; **IAP** – intracisternal A-type particle; **IFA** – indirect fluorescent antibody; **IHC** – immunohistochemistry; **MLV/MuLV** – murine leukemia virus; **MLRV-** murine leukemia virus-related virus; **MS** – multiple sclerosis; **mt** – mitochondrial; **PBMC/PBMNCs** – peripheral blood mononuclear cells; **PCA** – prostate cancer; **SFFV** – spleen focus**-**forming virus; **TMA** – transcription mediated amplification; **XMRV** – xenotropic murine leukemia virus-related virus.
